# Post-COVID-19 vaccine-associated menstrual cycle changes: A multifaceted problem for Pakistan

**DOI:** 10.1016/j.amsu.2022.103774

**Published:** 2022-05-11

**Authors:** Ayesha Liaquat, Zunera Huda, Saleha Azeem, Hala Najeeb

**Affiliations:** a Department of Internal Medicine, Dow Medical College, Dow University of Health Sciences, Baba-e-Urdu Road, Saddar, Karachi, Pakistan; b Department of Internal Medicine, King Edward Medical University, Nila Gumbad Chowk, Neela Gumbad, Lahore, Pakistan

## 1 Main text

Last year, the National Institute of Health (NIH) agreed to fund five institutes to investigate a possible association between the coronavirus disease 2019 (COVID-19) vaccination and the menstrual cycle, with its associated pathophysiology [1]. The need for this initiative arose when approximately 36,000 women reported abnormal vaginal bleeding or menstrual alterations such as heavier flow and delayed periods post-COVID-19 vaccination [2]. Moreover, a recent U.S. cohort study has reported a significant association between COVID-19 vaccination and slight alterations in menstrual cycle length; however, no change was observed in the duration of menses [3]. Although the exact pathophysiology has not been well established, several studies have attributed these menstrual changes to vaccine-induced thrombotic thrombocytopenia (VITT). As highlighted by Saleem et al., VITT results in losing endometrial hemostasis, which manifests as heavy menstrual bleeding [4].

Unfortunately, in Pakistan where social media platforms such as Twitter and Facebook brim with women who experienced menstrual disturbances post-COVID-19 vaccination, newspapers and scientific journals remain vacant in this regard. Thus, this lack of empirical evidence and concern has pushed us to highlight a long-standing problem of neglecting menstrual health in the Pakistani healthcare system. Period poverty, defined as the inaccessibility of sanitary products and facilities, and a lack of knowledge and awareness of menstrual hygiene management (MHM), is highly prevalent in Pakistan [5]. It is especially highlighted in rural and remote areas of Pakistan, where menstruation results in school absenteeism due to the unavailability of sanitary facilities [6]. In areas where availability is not an issue, affordability presents another problem. The government imposes hefty taxes on sanitary products and classifies them as ‘non-essential’ and ‘luxury’ items, contrary to men's hygiene products that are ‘essential’ [7]. Therefore, women are forced to use unhygienic absorbent materials like rags, and cotton, which are reused and shared, consequently leading to genital tract infections [8]. Apart from this, socio-cultural norms prove to be another major impediment to combating period poverty. The menstrual taboo is deep-rooted; a menstruating woman is considered unclean and impure, with an intense shame associated with open dialogue where menstruation is concerned [9]. Consequently, there exists a considerable population of women who has negligible knowledge about this natural biological process and its management. In a survey-based study conducted in Quetta, Balochistan, 22.4% and 15.2% of the female participants were uninformed about menstruation and its cause, respectively, and 77.7% had never received any information about it in school [6]. Similarly, a cross-sectional study conducted in Karachi, a metropolitan city, showed that most participants had little to no knowledge of sanitary products such as tampons and menstrual cups. In the same study, it was revealed that only 32.2% of healthcare workers and 29% of the general populace who experienced menstrual irregularities consulted a doctor [10].

Therefore, it is imperative that the Pakistani government and the general population, specifically women, acknowledge the lack of awareness of menstrual health to mitigate the prevailing COVID-19 vaccine hesitancy in Pakistan. The reluctance to get the vaccine most commonly stems from the fear of unspecified adverse effects [11]. Since post-COVID-19 menstrual disturbances are one of such side effects, physicians must inform females about possible menstrual changes, especially if the patient suffers from any platelet disorders. This will help cater to the mistrusts and doubts associated with COVID-19 vaccines. Vaccinated women should also be encouraged to log these menstrual changes into apps like *“Flo”* and “*Period Tracker*”. Parallel to Edelman et al., data from such apps can be recruited and analyzed in future studies targeting mainly South Asian females.

However, besides these temporary actions, some fundamental changes need to be made to tackle the problem at the grassroots level. The government has tried to overcome the menstruation taboo and period poverty by taking multiple initiatives, such as National Reproductive Health Services Package, Lady Health Worker Program, alongside endorsing WHO-associated projects like “Ao Baat Karein” and “The Puberty Book” however, they are not sufficient. Pakistan could adopt Scotland's example and provide sanitary napkins with minimal taxes or free of cost at all educational institutes and workplaces nationwide [5]. Additionally, for most girls, the primary sources of information about menstruation are their mothers [6], who are ill-informed. Therefore, menstrual education must be made compulsory in the school curriculum, like in England, to prevent the spread of menstrual misinformation, create awareness and destigmatize the subject of menstruation. Furthermore, NGOs like *Sehat Kahani, Aahung, and Marie Stopes Society* should be subsidized and promoted by the government on a national level. Sehat Kahani carried out a successful hybrid telehealth case study in collaboration with Ipas Pakistan, the Punjab Department of Health, and a network of Lady Health Workers (LHWs) in Islamabad and several districts of Punjab during the pandemic where gynecological e-consultations were provided; the project was a success [12]. Hence, telemedicine has much potential in making sexual and reproductive health services available in rural and remote areas with adequate government support in providing the necessary equipment. In conclusion, women's reproductive, specifically menstrual health, has been ignored for far too long; it is not only fair but also pertinent that it comes to the forefront now.

## Ethical approval

This paper did not involve patients, therefore no ethical approval was required.

## Sources of funding

No funding was acquired for this paper.

## Author contribution

Ayesha Liaquat: conception of the study, major drafting of the work, literature search, final approval and agreeing to the accuracy of the work. Zunera Huda: conception of the study, literature search, major drafting of the work, final approval and agreeing to the accuracy of the work. Saleha Azeem: conception of the study, major drafting of the work, final approval and agreeing to the accuracy of the work. Hala Najeeb: conception of the study, drafting of the work, final approval and agreeing to the accuracy of the work.

## Guarantor

Ayesha Liaquat, Zunera Huda, Saleha Azeem, Hala Najeeb.

## Consent

This study was not done on patients or volunteers, therefore no written consent was required.

## Declaration of competing interest

The authors declare that there is no conflict of interest.
